# The Kinesin AtPSS1 Promotes Synapsis and is Required for Proper Crossover Distribution in Meiosis

**DOI:** 10.1371/journal.pgen.1004674

**Published:** 2014-10-16

**Authors:** Yann Duroc, Afef Lemhemdi, Cécile Larchevêque, Aurélie Hurel, Maria Cuacos, Laurence Cromer, Christine Horlow, Susan J. Armstrong, Liudmila Chelysheva, Raphael Mercier

**Affiliations:** 1 The French National Institute for Agricultural Research (INRA), Institut Jean-Pierre Bourgin, UMR 1318, ERL CNRS 3559, Saclay Plant Sciences, RD10, Versailles, France; 2 AgroParisTech, Institut Jean-Pierre Bourgin, UMR 1318, ERL CNRS 3559, Saclay Plant Sciences, RD10, Versailles, France; 3 School of Biosciences, University of Birmingham, Birmingham, United Kingdom; University of Cambridge, United Kingdom

## Abstract

Meiotic crossovers (COs) shape genetic diversity by mixing homologous chromosomes at each generation. CO distribution is a highly regulated process. CO assurance forces the occurrence of at least one obligatory CO per chromosome pair, CO homeostasis smoothes out the number of COs when faced with variation in precursor number and CO interference keeps multiple COs away from each other along a chromosome. In several organisms, it has been shown that cytoskeleton forces are transduced to the meiotic nucleus via KASH- and SUN-domain proteins, to promote chromosome synapsis and recombination. Here we show that the *Arabidopsis* kinesin AtPSS1 plays a major role in chromosome synapsis and regulation of CO distribution. In *Atpss1* meiotic cells, chromosome axes and DNA double strand breaks (DSBs) appear to form normally but only a variable portion of the genome synapses and is competent for CO formation. Some chromosomes fail to form the obligatory CO, while there is an increased CO density in competent regions. However, the total number of COs per cell is unaffected. We further show that the kinesin motor domain of AtPSS1 is required for its meiotic function, and that AtPSS1 interacts directly with WIP1 and WIP2, two KASH-domain proteins. Finally, meiocytes missing AtPSS1 and/or SUN proteins show similar meiotic defects suggesting that AtPSS1 and SUNs act in the same pathway. This suggests that forces produced by the AtPSS1 kinesin and transduced by WIPs/SUNs, are required to authorize complete synapsis and regulate maturation of recombination intermediates into COs. We suggest that a form of homeostasis applies, which maintains the total number of COs per cell even if only a part of the genome is competent for CO formation.

## Author Summary

In species that reproduce sexually, diploid individuals have two copies of each chromosome, inherited from their father and mother. During a special cell division called meiosis, these two sets of chromosomes are mixed by homologous recombination to give genetically unique chromosomes that will be transmitted to the next generation. Homologous recombination processes are highly controlled in terms of number and localization of events within and among chromosomes. Disruption of this control (a lack of or improper positioning of homologous recombination events) causes deleterious chromosome associations in the offspring. Using the model plant *Arabidopsis thaliana* we reveal here that the *AtPSS1* gene is required for proper localization of these homologous recombination events along the genome. We also show that AtPSS1, which belongs to a family of proteins able to move along the cytoskeleton, is likely part of a module that allows cytoplasmic forces to be transmitted through the nucleus envelope to promote chromosome movements during homologous recombination progression.

## Introduction

During meiosis, chromosomes inherited from the mother and father are mixed in a process termed homologous recombination, to generate unique chromosomes that will be transmitted to the next generation. This genetic mixing has sustained the evolution of eukaryotes. There are typically one to four exchange points –crossovers (COs)- between homologous chromosomes at each meiosis. The distribution of these COs is under a series of constraints [1], [2]. First, there is at least one CO per chromosome pair (obligatory CO or CO assurance). Indeed, beyond their genetic consequences, COs are also essential for holding homologous chromosomes together during meiosis I, ensuring their balanced distribution in daughter cells. Notably, a lack of or improper positioning of this obligatory CO causes aneuploidy in human oocytes [3]. Second, COs are subject to interference. This prevents the occurrence of COs next to each other, shaping an even distribution and limiting their number [4]. COs are also under homeostasis, meaning that their number tends to be stable even when faced with variation in precursor number [5]–[7]. Finally, looking at frequencies, COs are not homogenously distributed along the genome; hot and cold regions have been defined at the chromosome scale, and hotspots with a very high CO frequency have been observed at the kb scale [8], [9].

COs are produced during meiotic prophase I concomitantly with and functionally connected to chromosome pairing and synapsis, which is the intimate association of homologous chromosomes lengthways with a protein structure, the synaptonemal complex (SC). Recombination is initiated at early prophase I by the formation of DNA double-strand breaks (DSBs) which largely outnumber the eventual CO number [10]. DSBs are subsequently resected to yield 3′ overhangs that invade the homologous chromosome, a step in which the recombinase DMC1 plays a prominent role [11]. In plants, as in mammals and budding yeast, these early steps of recombination also promote homologous chromosome synapsis. Indeed mutants affected in DSB formation or homologous template invasion (including *Atdmc1*) fail in both synapsis and CO formation [12]–[14]. DSB repair events form intermediates that are eventually resolved as either COs or non-crossovers (NCO) [15], [16] in the context of the SC. DSB repair can also occur using the sister as a template, a process that does not lead to inter-homologue COs, as observed in the *Atdmc1* mutant [12], [13] or during haploid meiosis [17] where the ubiquitous recombinase RAD51 catalyzes sister repair. However the prevalence of such sister-mediated repair in wild-type *Arabidopsis* is unclear [15]. Homologous chromosome invasion events can mature into COs through at least two independent pathways. These two pathways coexist in budding yeast, mammals and *Arabidopsis* [1], [18]–[21]. Class I COs, the most prevalent class, are subject to interference and their production is dependent on the ZMM proteins (in *Arabidopsis*: SHOC1 (AtZIP2), PTD1, AtHEI10, AtZIP4, AtMSH4, AtMSH5, AtMER3 [18]). Interestingly, in *Arabidopsis zmm* mutants most chromosomes do not form COs, but synapsis occurs normally. This shows that recombination intermediates which promote synapsis are produced in *zmm* mutants, even if they are not eventually converted into COs. Formation of the minor Class II COs (∼15% of CO), that do not display interference, involves MUS81 [1], [18]–[21] and is down regulated by AtFANCM [22], [23].

All these molecular events must be coordinated at the chromosome and cellular levels to shape CO distribution. Interestingly, it has been shown in several species that chromosome movement is particularly prominent at meiotic prophase and plays a significant role in chromosome pairing/synapsis and CO formation [24], [25]. Telomeres in mammals, fungi and plants, or specific chromosome sites called pairing centers in the case of *C. elegans*, bind to the nuclear envelope where they are subject to cytoskeleton originated forces [26]–[31]. Telomere chromosome movements are also illustrated by a transient prophase I configuration called the bouquet, where telomeres cluster together on the nuclear envelope. This highly polarized nucleus stage has been described since the early 1900s [32]. Cytoplasmic forces are transduced to the chromosomes inside the nucleus, through the nuclear envelop which is intact during meiotic prophase I, *via* a chain of proteins (reviewed in [24], [25]). Central in this chain are the KASH (Klarsicht/ANC-1/Syne-1 homology)-domain and SUN(Sad-1/UNC-84)-domain proteins. In yeasts, worms and mammals KASH-domain and SUN-domain proteins play a crucial role in meiotic chromosome movement and homologue pairing [25], [27], [29], [33], [34]. KASH-domain proteins localize to the outer nuclear membrane and interact with SUN-domain proteins which are inserted in the inner membrane. However, the connection to the cytoskeleton, at one end, and to the chromosome (telomeres in most species and pairing centers in *C. elegans*) associated protein at the other end, appears to rely on evolutionary divergent proteins (reviewed in [24], [25]). In *S. cerevisiae* KASH-domain proteins interact with actin [35] while *S. pombe, C. elegans* and mouse KASH-domain proteins interact with dynein [25], a microtubule motor protein. In plants, the movement of chromosomes at meiotic prophase has been directly observed in maize and the application of specific depolymerizing drugs suggests that it depends on both tubulin and actin [31]. In *Arabidopsis*, where live imaging is not yet available, telomeres appear to be associated around the nucleolus in early meiotic prophase and are moved to the nuclear membrane preceding synapsis where they transiently cluster together (but not in a tight manner as in the classical bouquet configuration observed in many species [36]). On completion of synapsis the paired telomeres are dispersed but remain attached to the nuclear membrane until diplotene when they dissociate from the nuclear membrane [37]. Thus, it is likely that telomere-mediated chromosome movement is also important for meiotic prophase I in *Arabidopsis*. Strongly supporting this hypothesis, the two *Arabidopsis* SUN-domain proteins were recently shown to be essential for completion of synapsis and normal CO formation (S.J.A, unpublished data).

The rice Kinesin1-like protein PSS1 has been shown to be essential for fertility and normal chromosome segregation at meiosis [38], but its potential function in synapsis and recombination was not investigated. Here we identified the AtPSS1 Kinesin1-like protein as a major actor in meiosis, promoting synapsis and regulating CO formation in *Arabidopsis*. Our data suggest that the movement of AtPSS1 along microtubules generates cytoplasmic forces which could be transmitted to the chromosomes via a KASH-SUN module and coordinate synapsis and CO distribution.

## Results

### AtPSS1 is required for full synapsis and bivalent formation in meiosis

**Figure 1:**
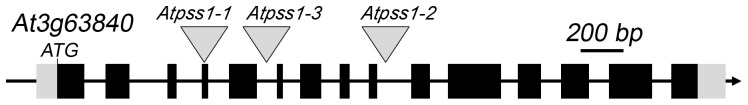
The *AtPSS1* gene and mutations. The arrow indicates the orientation of the open reading frame. Exons are shown as boxes (grey: UTR, black: CDS). In *Atpss1-1*, *Atpss1-2* and *Atpss1-3* corresponding to WiscDsLox_343E05, SALK_120399 and SALK_024926 lines, the T-DNA was inserted as indicated by triangles.

A previous report showed that mutation of the rice class I kinesin I (named OsPSS1) leads to meiotic defects [38]. Reciprocal BLAST analysis and comprehensive sequence analysis of plant kinesins [39] unambiguously identified the product encoded by the *Arabidopsis* At3g63480 gene as the only putative orthologue of *OsPSS1*. The two proteins share high amino acid sequence identity (59%). We identified three T-DNA insertion lines from the public collections: *Atpss1-1*, *Atpss1-2* and *Atpss1-3*. Insertion of the TDNA in these loci was confirmed by sequencing the flanking sequences (Figure 1). Homozygous plants for all three lines have the same phenotype: normal vegetative growth but decreased fertility, as shown by reduced seed set (55±6 seeds per silique for wild type versus 27±5 for *Atpss1-1*) and reduced pollen viability (Alexander staining, Figure S1). Heterozygote plants for two *Atpss1* mutations had the same phenotype showing that the three mutants are allelic. Transformation of the *Atpss1-1* mutant with a 5 kb genomic region containing the *AtPSS1* coding and regulatory sequences restored pollen viability (7 independent transformants, Figure S1), confirming that the observed defects are due to disruption of the *AtPSS1* gene.

**Figure 2:**
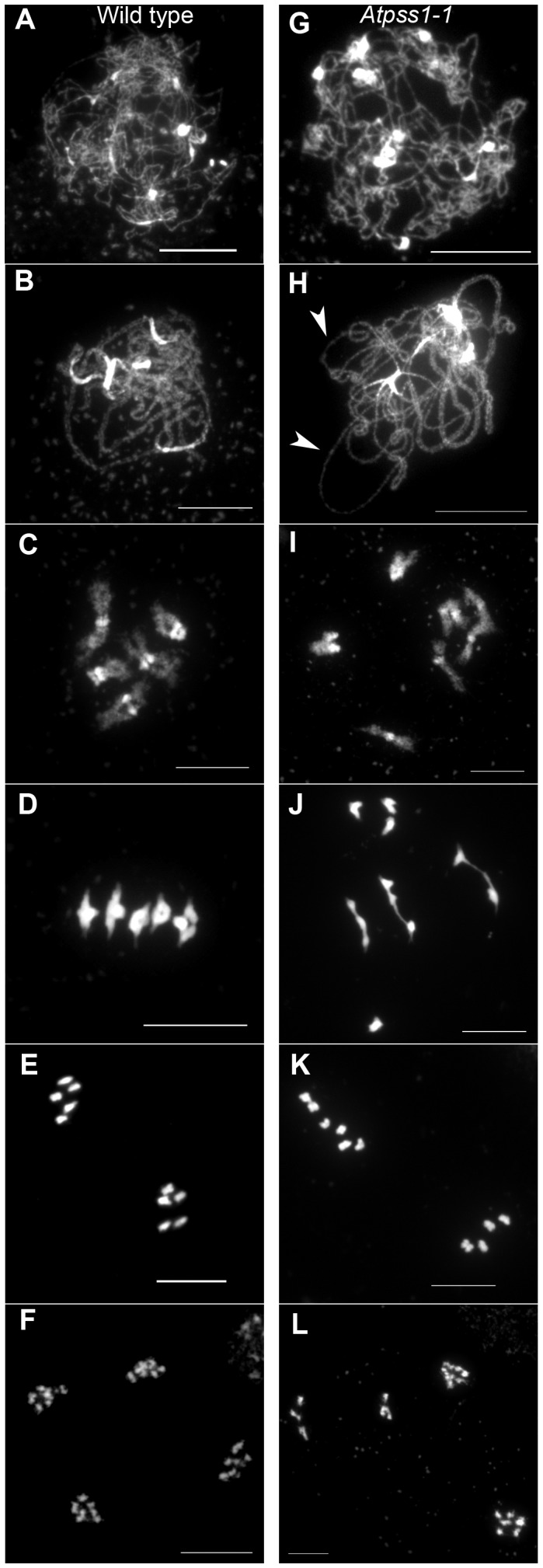
Chromosome spreads of male meiocytes in wild type and *Atpss1-1*. (A,G) Leptotene. (B,H) Pachytene. Arrowheads show unsynapsed regions. (C,I) Diakinesis. (D,J) Metaphase I. (E,K) Metaphase II. (F,L) Telophase II. Chromosome were spread according to Ross *et al.* [40] and stained with DAPI. Scale bar = 10 µm.

**Figure 3:**
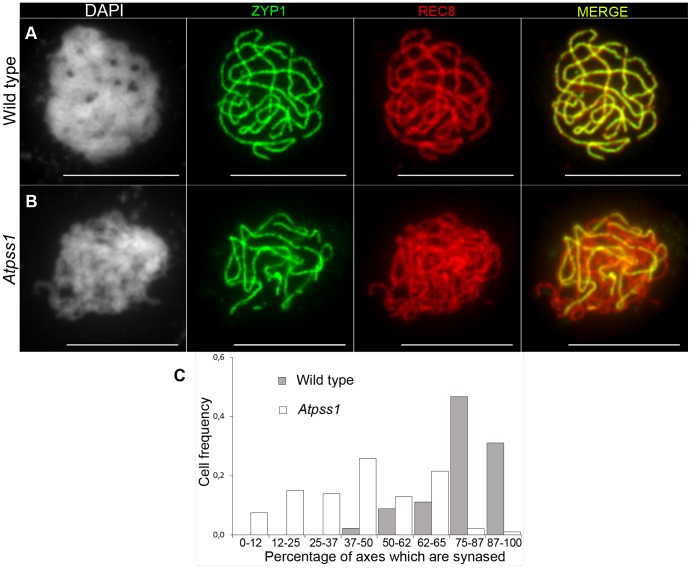
Co-immunolocalization of REC8 and ZYP1 at pachytene. Floral buds of the correct size or bigger for the late pachytene/diplotene stage in wild type were used to make spreads according to Armstrong *et al.* [42]. Scale bar = 10 µm. (A) Wild type. (B) *Atpss1-1*. (C) Histogram of cells according to their proportion of synapsed axes. The proportion of synapsed axes in each cell was estimated by measuring the frequency of [red and green] pixels among the total number of [red] pixels. For example on figure 3B, 51% of the ASY1 red signal (axes) colocalize with the ZYP1 green (synapsis) signal.

**Figure 4:**
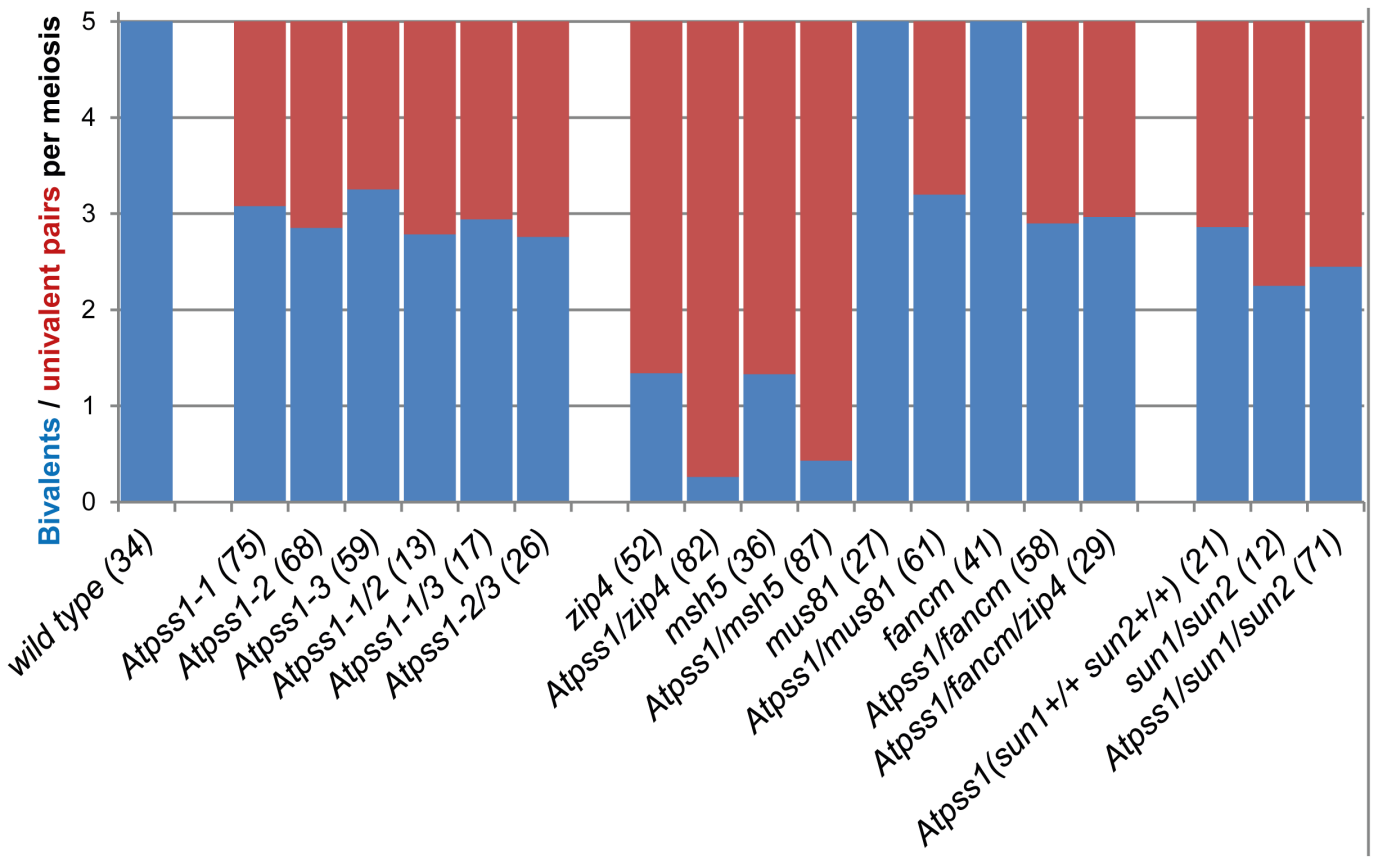
Average number of bivalents (blue) and pairs of univalents (red) per male meiocyte. Number of metaphase I cells analyzed is indicated in brackets.

**Figure 5:**
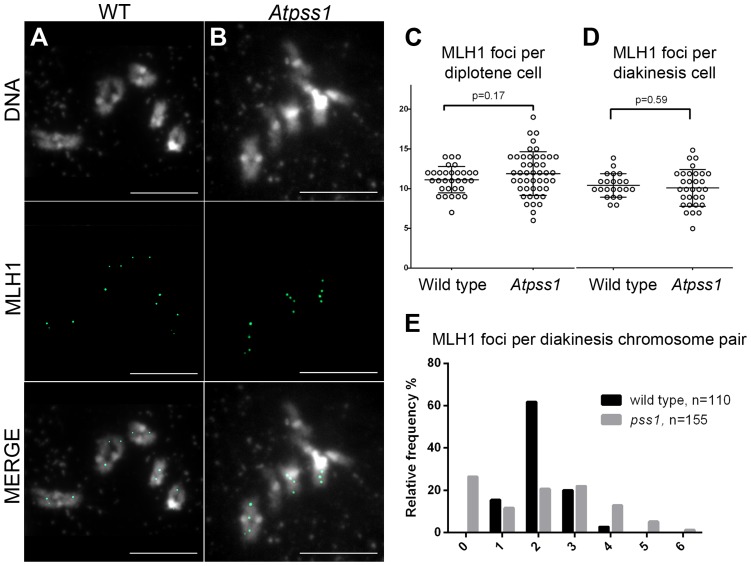
MLH1 immunolocalization. Immunolocalization of MLH1 at diakinesis is shown (A) in wild type and (B) in *Atpss1-1*. (C, D). Scatter plot of MLH1 foci number per cell at diplotene and diakinesis. (E) Distribution of chromosomes according to their MLH1 foci number at diakinesis. Cells were prepared according to Chelysheva *et al.* [47]. Scale bar = 10 µm.

We used chromosome spreads to investigate male meiosis defects in the *Atpss1-1* mutant. Wild-type *Arabidopsis* meiosis was described in detail in [40], and the major stages are summarized in figure 2. At leptotene chromosomes appear as thin threads (Figure 2A), synapsis (the close association of two chromosomes via an SC) begins at zygotene and is complete by pachytene (Figure 2B). The SC is then depolymerized at diplotene and chromosomes condense so that the five bivalents are visible (pairs of homologues connected by COs) (Figure 2C). The bivalents align at metaphase I (Figure 2D), and chromosomes separate from their homologue at anaphase I leading to the formation of two pools of five chromosomes and two nuclei (Figure 2E). At the second meiotic division, the pairs of sister chromatids align on the two metaphase plates, and separate at anaphase II to generate four pools of five chromosomes, which gives rise to tetrads of four microspores (Figure 2F). In *Atpss1* mutants, leptotene and zygotene appeared similar to those in wild type (Compare figure 2A and 2G). Accordingly immunolocalization of two axial element proteins, ASY3 [41] and the hormad domain containing protein ASY1 [42] did not reveal any difference between *Atpss1* and wild type (Figure S2). However we were unable to find a typical pachytene stage among chromosome spreads of the *Atpss1* mutant (n>300), as only partial synapsis was observed (Figure 2H). Synapsis was further examined by immunolocalization of REC8 and ZYP1 [43], which are chromosome axis and SC central element proteins, respectively (Figure 3). In flower buds whose size corresponds to late pachytene/diplotene stages, most wild-type cells showed almost complete synapsis, with the ZYP1 signal covering completely the REC8 signal (Figure 3A). In contrast, we were unable to find any meiocytes in which SC had undergone complete polymerization (n = 102) in At*pss1*. At*pss1* cells showed various levels of incomplete synapsis (Figure 3B), ranging from 4 to 91%, with less than half of the REC8 axis being covered with ZYP1 signal in most cells (distribution shown on Figure 3C). The observed partial ZYP1 loading could be the result of either delayed synapsis or failure in completing synapsis. However, the observation of diplotene stages on the same slides favors the hypothesis of incomplete synapsis (see also below). At diakinesis and metaphase I, a mixture of univalents and bivalents (on average 3.1±1.2 bivalents and 1.9±1.2 univalent pairs) was observed in each *Atpss1* allele (Figure 4), contrasting with wild type which always has five bivalents (Figure 2D, J and 5). FISH experiments using probes directed against 45S, 5S rDNA and the F8J2 BAC that allow the identification of the five *Arabidopsis* Col-0 chromosomes as described in [44], suggested that each chromosome is affected in bivalent formation (The univalent frequency for chromosomes 1 to 5 were respectively 28%, 37%, 42%, 42% and 26%. N = 43 *Atpss1-1* cells). The presence of univalents resulted in missegregation of chromosomes in anaphase I and a subsequent aberrant number of daughter cells and/or unbalanced chromosome distribution (Figure 2K, L). Overall, our results showed that AtPSS1 is required for full synapsis and normal levels of bivalent formation at male meiosis. Observation of pistils [45], showed that 52% of the *Atpss1* female gametophytes were defectives (n = 150). Further, univalents were detected at metaphase I of female meiosis (Figure S3), showing that AtPSS1 is essential for normal levels of bivalent formation in both male and female meiocytes.

### The *AtPSS1* mutation affects CO distribution but not frequency

**Figure 6:**
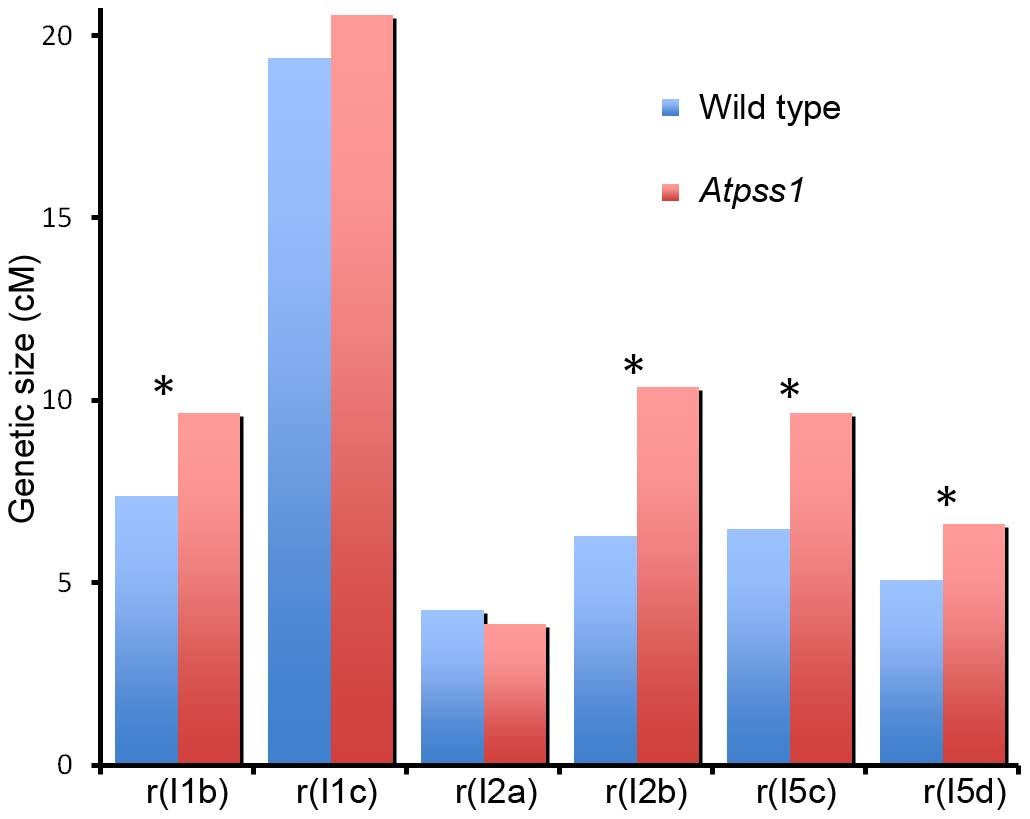
Genetic recombination in wild type and *Atpss1-1*. Genetic distances in six intervals using tetrad analysis with fluorescent-tagged lines (FTL), were calculated with the Perkins equation [67] and are given in centiMorgans. I1b and I1c are adjacent intervals on chromosome 1 and so on for the other pairs of intervals as described in [46] (Table S1).

The presence of bivalents in *Atpss1-1* implies that CO formation is not completely impaired in this mutant. The nature of the COs produced in the absence of AtPSS1 was investigated by epistasis tests with *zmm* and *mus81* mutants, which are defective in class I and class II CO formation, respectively. Mutation of a *ZMM* in *Atpss1* reduced bivalent formation from 3.1±1.2 to 0.3±0.4, showing that most of the COs produced in the *Atpss1* mutant are ZMM dependent. We then used MLH1 immunolocalization, a marker of class I COs, to explore CO distribution in *Atpss1*. The total number of MLH1 foci per cell during diplotene and diakinesis was similar in *Atpss1* (11.9±2.7 and 10.2±2.3) and wild type (11.1±1.7and 10.5±1.5) (Figure 5). However, we found that the distribution of MLH1 foci among chromosomes was significantly affected in the *Atpss1* mutant, as shown in figure 5. In wild type, 62% of the bivalents had exactly two MLH1 foci, 20% had three, 15% had one and less than 3% had four foci. In contrast, the number of MLH1 foci per chromosome was much more variable in *Atpss1*, with the appearance of classes not observed in wild type (Figure 5E). One quarter of the chromosome pairs appeared as univalents without MLH1 foci, fitting with the observed frequency of univalents at metaphase I, while bivalents with more than three foci were more frequent than in wild type (19.4% vs 2.7%). This suggests that CO distribution but not frequency is affected in *Atpss1*. Measurements of recombination rates in six genetic intervals using pollen tetrad analysis [46] showed that CO frequency is not reduced but even slightly higher in *Atpss1* (Figure 6, Table S1a and Table S1b). CO interference, measured genetically, was significantly reduced compared to wild type, to a level no longer detected (Table S1b). While we cannot formally exclude that a low level of interference exists, this clearly establish that CO interference measured genetically is decreased in *Atpss1*. This further suggests that relative CO distribution is disturbed in *Atpss1*.

### In *Atpss1*, synapsis and DSBs maturation into COs occur in the same regions

**Figure 7:**
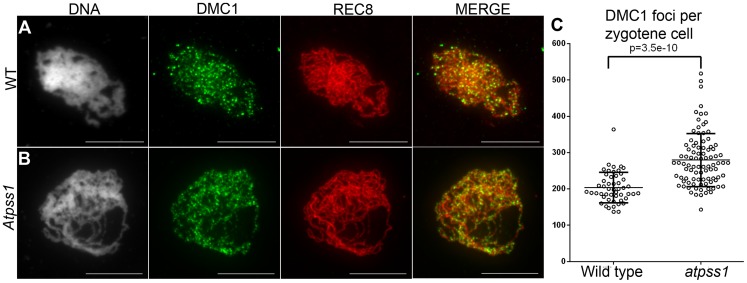
DMC1 immunolocalization. Immunolocalization of DMC1 at early zygotene is shown (A) in wild type and (B) in *Atpss1-1*. Cells were prepared according to Armstrong *et al*. [42] Scale bar = 10 µm. (C) Scatter plot of DMC1 foci number per cell.

**Figure 8:**
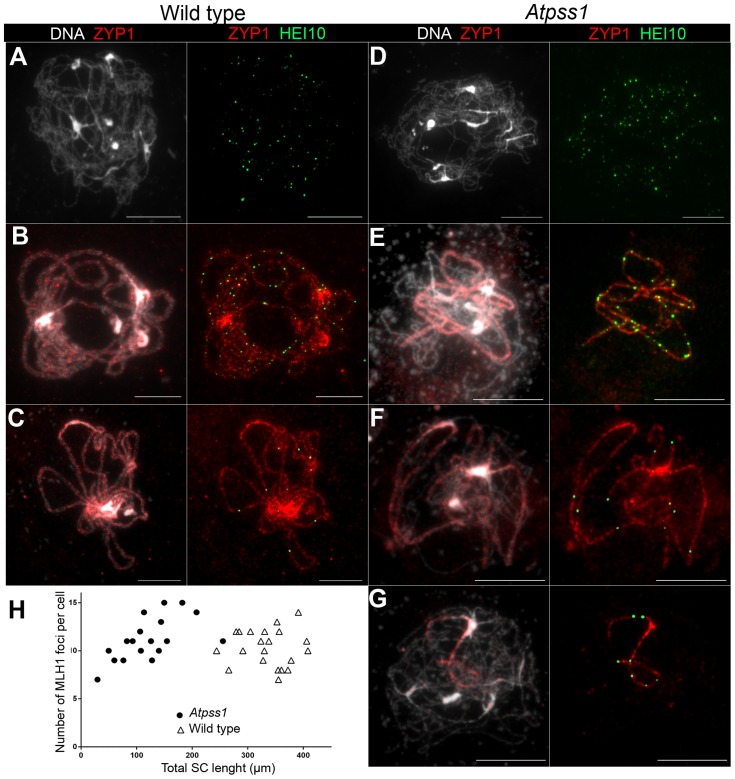
Co-immunolocalization of HEI10 and ZYP1. (A, D) Leptotene with numerous HEI10 foci, (B, E). Early pachytene with a mixture of faint and bright HEI10 foci. (C, F, G) Late pachytene with bright HEI10 foci. (H) Plot of pachytene cells according to their total HEI10 foci number and total SC length. Cells were prepared according to Chelysheva et al. [47] Scale bar = 10 µm.

Overall, the above data showed that synapsis is incomplete and CO distribution among chromosomes is affected in *Atpss1* mutants. As both synapsis and COs are promoted by DSB formation and repair, we carried out immunolocalization studies with DMC1, a protein which marks DSBs undergoing repair. In *Atpss1*, DMC1 foci decorated all chromosome axes and their total number was higher compared to wild type (+37%. 204±6 vs 279±8. T-test p = 3.5.10^−10^), suggesting that in the mutant DSB formation is enhanced or that DMC1 foci accumulate due to slower turnover (Figure 7). Thus in the mutant DSBs appear to occur on all chromosomes. We then examined whether the chromosome regions where COs occurred and that synapsed were the same. Because synapsis disappears before MLH1 foci numbers peak in *Arabidopsis* [47], we used HEI10/ZYP1 co-immunolocalization to explore this question (Figure 8 and S4). Indeed, HEI10 marks recombination progression from numerous faint foci at leptotene (Figure 8A) to about ten large foci labeling class I CO sites from late pachytene (Figure 8C) to diakinesis (Figure S4) [48]. At leptotene, *Atpss1* and wild-type cells were indistinguishable with numerous small HEI10 foci (Figure 8A and 8D), further suggesting that early recombination events are unaffected in the mutant. At early wild type pachytene, numerous foci of variable size are dispersed on the SC (Figure 8B). At the same stage in *Atpss1*, the synapsed regions were also decorated with numerous HEI10 foci, but the regions that failed to synapse were foci-free. At late pachytene, a small number of bright and homogeneous foci were observed in both wild type and the mutant (Figure 8C, F and G). Remarkably, while the total length of the SC in *Atpss1* pachytene cells was on average one third that of wild type, confirming partial synapsis, the average number of HEI10 foci per cell was unaffected (Wild type: 10.3±1.9, *Atpss1*: 11.2±1.2, p = 0.19) (Figure 8H). Accordingly, the number of HEI10 foci per 100 µm of SC was on average 3.1±0.7 for wild type and 10.9±4.8 for *Atpss1* (these measurements were made on a cell per cell basis, because the entanglement of *Arabidopsis* pachytene chromosomes makes it difficult to unambiguously follow individual SCs). While the density of HEI10 foci was relatively stable in wild type (from 2 to 4.3 per 100 µm), it varied greatly in *Atpss1* (from 4.3 to 23.6 per 100 µm) (Figure 8H). This is strikingly illustrated by the extreme case shown in figure 8G, where seven HEI10 foci can be seen on a single 30 µm SC stretch. At diplotene and diakinesis, the number of HEI10 foci per cell was similar and stable in the wild type and mutant. However, consistent with the MLH1 data, the distribution of HEI10 foci among chromosomes was significantly modified in *Atpss1* (Figure S4), confirming that CO distribution but not number is affected. In summary, in *Atpss1*, COs and synapsis are jointly restricted to the same limited portion of the genome. Partial synapsis is accompanied by an increase in CO density per SC unit, resulting in –or caused by (see discussion)- an unaffected number of COs per cell.

### MUS81-dependant COs in *Atpss1*

The *MUS81* pathway (Class II pathway) is minor in *Arabidopsis* wild type. Its disruption reduces CO frequency by ∼10%, but does not affect bivalent formation [49], [50] (Figure 4). Mutation of *MUS81* in the *Atpss1* background did not further reduce bivalent frequency (Figure 4), which is consistent with the conclusion above that most COs are ZMM-dependent in *Atpss1*. At *FANCM* was previously shown to limit MUS81-dependant CO formation and bivalent formation is fully restored in *zmm/Atfancm* mutants due to a massive increase in class II COs [22]. Mutation of *AtFANCM* in *Atpss1* did not increase the number of bivalents, suggesting that it did not restore CO formation in regions that are CO incompetent in the single *Atpss1* mutant (but this does not exclude that there is an increase in CO frequency in regions that are CO competent) (Figure 4). However while bivalent formation was very low in *Atpss1 Atzip4*, bivalent formation was restored in the *Atpss1 Atfancm Atzip4* triple mutant back to the level observed in the single *Atpss1* mutant (Figure 4). Altogether, these results suggest that, in *Atpss1*, class II COs occur at a low frequency, and can be promoted by mutating *AtFANCM* but exclusively in regions that are also already competent for class I CO formation.

### A potential AtPSS1-SUNs-WIPs force transduction module

**Figure 9:**
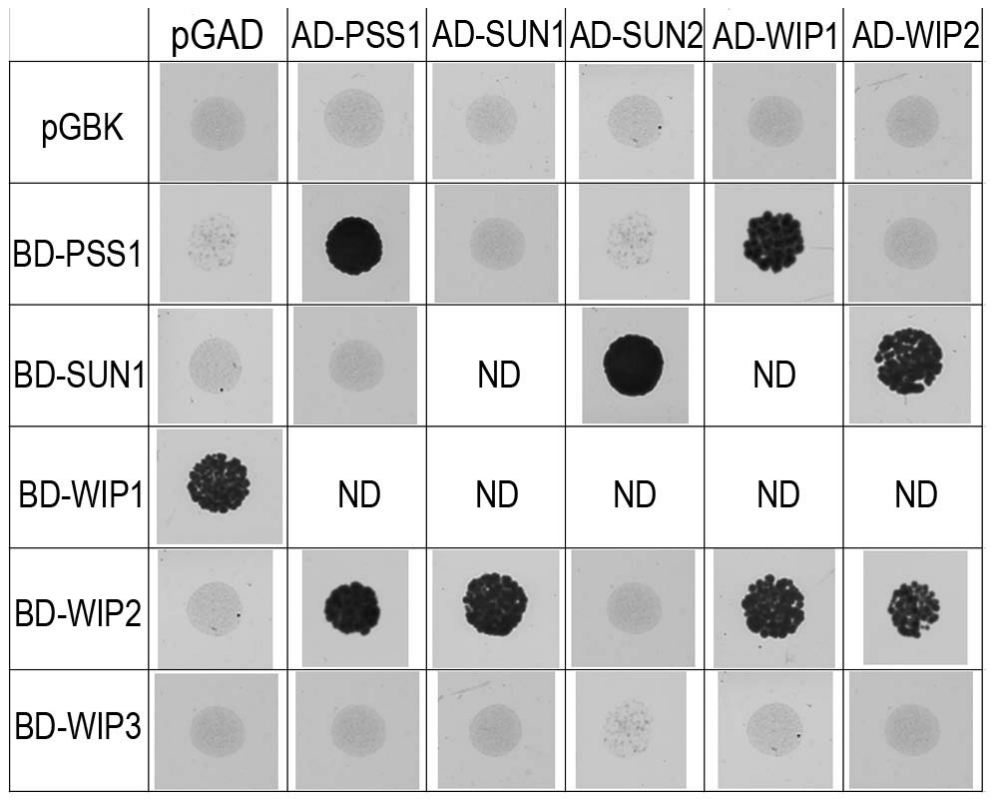
Interaction between AtPSS1, AtWIPs and AtSUNs. Yeast two-hybrid - For each combination yeast cells were spotted on selective medium to test interactions (all combinations were able to grow on non-selective medium; not shown) ND: Not determined either because irrelevant or due to self-activation of one of the partners.

AtPSS1, which belongs to the kinesin family, appears to play a crucial role in meiosis. Kinesin proteins are characterized by their ability to walk on microtubules via a motor domain that uses ATP to promote repetitive conformation changes [51]. We thus tested if the motor function of AtPSS1 is important for its function in meiosis. For this, we expressed an AtPSS1 protein modified in the conserved arginine (Arg-293>His) that was previously shown to abolish the microtubule-stimulated ATPase activity [38] in the *Atpss1-1* mutant. When *Atpss1* plants were transformed with the control wild-type *AtPSS1* gene, pollen viability and bivalent formation at metaphase I were fully restored (7 independent transformants). In contrast, transformation with *AtPSS1-R293H*, expressed behind the native *AtPSS1* promoter, did not restore pollen viability and normal meiosis (4 independent transformants, see methods; Bivalent frequency: 4.2±1 (n cells = 35), 4.3±0.5 (n = 6), 3.5±0.6 (n = 4), 3.5±1.2 (n = 15), respectively), showing that the kinesin function of AtPSS1 is critical for its role in meiosis. In several model species, cytoskeleton-based forces were previously shown to be important for meiosis and to be transduced to the nucleus by KASH- and SUN-domain containing proteins [24], [25]. In *Arabidopsis*, two SUN proteins were recently shown to be redundant and important for meiosis (S.J.A. under review). As in *Atpss1*, a mixture of bivalents and univalents are observed in *Atsun1 Atsun2* double mutants. This defect is quantitatively identical in the *Atpss1, Atsun1 Atsun2* and the *Atsun1 Atsun2 Atpss1* triple mutants (Figure 4), suggesting that SUN proteins and AtPSS1 may act in the same pathway. WIP1-3 proteins were also recently identified as KASH containing proteins in *Arabidopsis*, and shown to interact with SUNs [52]. This raised the possibility that AtPSS1 could be involved in transmitting forces to the meiotic nucleus via a WIP-SUN module. Yeast two-hybrid experiments showed that AtPSS1 interacts directly with WIP1 and WIP2. The AtPSS1-WIP1 but not the AtPSS1-WIP2 interaction was confirmed by BiFC assays (Figure S5). The yeast two-hybrid also confirmed that WIPs interact with SUNs, as previously shown [52] (Figure 9).

## Discussion

During meiotic prophase I, chromosome movements within the intact nucleus are prominent and have been shown to be involved in chromosome pairing, synapsis and recombination in a variety of species. Here we showed that AtPSS1, the *Arabidopsis* kinesin-1 like protein [39], is essential for full synapsis and is required for proper CO distribution. Furthermore, the bivalent shortage is identical when AtPSS1, SUNs or both, are knocked out suggesting that SUNs and AtPSS1 act in the same pathway to regulate CO formation. In addition, AtPSS1 interacts with the KASH-domain proteins WIP1 and WIP2 which themselves interact with SUN proteins [52]. Finally, we showed that the kinesin motor domain of AtPSS1 is required for its meiotic function. Kinesin is a motor protein which walks along microtubules with high processivity and for long distances (reviewed in [51]). We thus speculate that AtPSS1 moves along microtubules and generates forces that are transduced via a SUN-WIP module through the nuclear membrane to the chromosomes, promoting synapsis and regulating CO distribution (see below). The proteins that would connect SUNs to the chromosome telomeres remain to be identified. These results add to a growing amount of evidence showing that the transduction of cytoplasmic forces through the nuclear membrane is an important and conserved promoter of meiotic recombination. It should be noted here that the function we propose for AtPSS1 appears to be fulfilled by dynein in many organisms, and that dynein is absent from flowering plant genomes [53]. The rice PSS1 is also essential for normal meiosis [38]. Even though recombination and synapsis have not been extensively analyzed in the rice *Ospss1* mutants, univalent were observed at metaphase I, suggesting that the primarily defects may be similar to *Atpss1*. This suggests that the meiotic function of AtPSS1 is conserved among flowering plants.

We showed that AtPSS1 is required for full synapsis and normal CO formation. In most species, the search for homologous sequences by recombinase-coated 3′-ssDNA promotes both CO formation and homologous synapsis. Indeed, in *Arabidopsis* both COs and synapsis are absent in mutants affecting DSB formation, but also homologous sequence invasion (RAD51, DMC1 and their co-factors) [12], [14], [18]. This appears to be a cooperative process as multiple repair events are required for initiation and progression of synapsis [54], [55]. *Atpss1* mutants have a novel defect: in each cell, COs and synapsis take place on only a subset of the genome (which varies from 10 to 90%). Initial DSB formation and processing do not appear to be involved in these defects, as DMC1 foci and early HEI10 are present on all chromosomes in the mutant. The number of DMC1 foci was higher in the mutant than wild type, possibly reflecting a delay in recombination progression. The increased number of DMC1 foci may also reflect an increase of the number of DSBs in response to the downstream defects [56]. However, we suggest that only a subset of these DSBs is efficiently matured into potential CO precursors and promoters of synapsis. This is supported by the observation that the segments of chromosomes which were seen to synapse were also the places where early HEI10 foci progressively matured into intermediate and then late CO-marking-foci. This model implies that chromosome movement involving AtPSS1 is required to efficiently mature DMC1-coated-DSBs into CO/synapsis precursors. This movement could be simply required for the homology searching DNA “tentacle” [57] to reach the homologous chromosome which can be at some distance in the nucleus [58]. Alternatively, the movement may be required to resolve the entanglement/clutter/interlocking which likely arises from multiple chromosome pairing attempts in the limited space of the nucleus [55]. The DSBs present on the portions of chromosomes which failed to reach homologues are likely repaired using the sister chromatids as template, thus failing to promote synapsis and homologous CO. Such sister-mediated repair occurs genome-wide in haploid *Arabidopsis*, where DMC1-coated resected DSBs are repaired on the sister, or in diploid mutants where DMC1 or one of its partners is absent [12]–[14], [59].

One intriguing feature of the *Atpss1* mutant is that CO frequency per cell is not reduced, but instead the subparts of the chromosomes that do synapse and recombine make a similar total number of COs per cell as in wild type. This is strikingly shown in figure 8G, where a single SC stretch was formed in a cell on which seven class I COs occurred, while CO number rarely exceeds four on an entire wild-type chromosome. The smaller size of the competent regions appears to be compensated by an increased CO density, which implies that interference is no longer acting or that the distance at which interference spreads is reduced. Unfortunately, the difficulty in following individual SCs prevented us from cytologically measuring CO interference. The stable number of COs per cell in *Atpss1* could reflect a form of CO homeostasis, which is defined as the tendency to preserve CO number despite a variation in DSB number through a modulation of the probability for DSBs to become COs [5]. We suggest that such homoeostasis applies in the *Atpss1* mutant, and that the decrease in the number of CO-competent DSB is compensated for by an increased probability of the eligible DSB becoming a CO. It is possible that the total number of COs per cell is defined, and then ∼10 COs per cell occurs on licensed regions. However, the mechanism that would count the number of COs per cell remains elusive. Alternatively, we suggest that a feed-back loop could sense some unachieved event (e.g. the presence of chromosomes lacking COs, or incomplete synapsis), and then increase the propensity of precursors to be designated for CO. This feed-back loop would therefore modulate the parameters of interference (possibly by a progressive increase in CO-promoting mechanical stress or progressive increase in the sensitivity of precursors to this stress [60], [61]). Finally, AtPSS1 could have a dual function, on one hand promoting synapsis and recombination intermediate maturation, and on the other preventing an excess of COs on selected regions, both via chromosome movement [24].

## Materials and Methods

### Plant material

Col-0 lines were obtained from the collection of T-DNA mutants from the Salk Institute Genomic Analysis Laboratory (Columbia accession) (SIGnAL, http://signal.salk.edu/cgi-bin/tdnaexpress) and provided by NASC (http://nasc.nott.ac.uk/). Mutant alleles used in this study were: *Atmsh5-2* (SALK_026553) [62]; *Atzip4-2* (SALK_068052) [63]; *Atmus81-2* (SALK_107515) [49], [50], *Atfancm-1* [22]; *Atsun1* (SAIL_84_G10); *Atsun2* (FLAG_026E12). Details for all genotypes, primers used and PCR amplification conditions are shown in table S2.

Plants were cultivated in a greenhouse or growth chamber under the following conditions: photoperiod 16 h/day and 8 h/night; temperature 20°C day and night; humidity 70%.

### Genetic analyses

The six intervals tested in this study correspond to intervals I1b and I1c (both located at the top of chromosome 1), I2a and I2b (both located at the bottom of chromosome 2), and I5c and I5d (both located at the top of chromosome 5) described in [64]. Tetrad analyses were carried out as described in [46]. The resulting tetrad data (Table S1a) were analyzed using the Perkins mapping equation.

All double mutants were obtained by crossing plants, which were heterozygous for each mutation. The resulting hybrids were self-pollinated. PCR screening was then used to identify plants in the F2 progeny that were homozygous for both mutations.

### Antibodies

The anti-ASY1 polyclonal antibody was described by [42]. The anti-ZYP1 polyclonal antibody was described by [43]. The anti-DMC1 antibody was described in [63], the anti-MLH1 antibody in [47], and the anti-HEI10 in [48]. The anti-REC8 polyclonal antibody was described in [65].

### Microscopy

Chromosome spreads of male meiocytes were prepared and stained with DAPI as described in [40]. Chromosome spreads for immunocytology was performed according to [42]. Observations were made using a Leica (http://www.leica.com) DM RXA2 microscope or a Zeiss (http://www.zeiss.fr) Axio Imager 2 microscope; photographs were taken using a CoolSNAP HQ (Roper, http://www.roperscientific.com) camera driven by OpenLAB 4.0.4 software or a Zeiss camera AxioCam MR driven by Axiovision 4.7. All images were further processed with OpenLAB 4.0.4, Axiovision 4.7, or AdobePhotoshop 7.0 (http://www.adobe.com).

### Yeast two-hybrid and BIFC assays

The *AtPSS1, AtWIP1, AtWIP2, AtWIP3, AtSUN1 and AtSUN2* open reading frames were amplified from *Arabidopsis* cDNA clones (Columbia ecotype) using specific primers flanked by the AttB1 and AttB2 sites (Table S2), cloned into Gateway vector pDONR207 using BP recombination (Invitrogen), and sequenced. Expression vectors were obtained after LR recombination (Invitrogen) between these entry vectors and destination vectors (pGADT7-GW and pGBKT7-GW for Y2H, and pBiFP vectors for BIFC). Yeast two-hybrid interactions were tested using AtPSS1, AtWIP1, ATWIP2, AtWIP3, AtSUN1 and AtSUN2 as bait (pGADT7-GW) or as prey (pGBKT7-GW) by mating with the AH109 and Y187 yeast strains. For fluorescence complementation tests, transient expression of all eight compatible combinations between protein pairs (i.e., providing both parts of the YFP) was assayed. Each expression vector was introduced into *Agrobacterium tumefaciens* strain C58C1(pMP90) by electroporation. *Agrobacterium* bacterial cultures were incubated overnight at 28°C with agitation. Each culture was pelleted, washed, and resuspended in infiltration buffer (13 g/L bouturage N°2 medium [Duchefa Biochemie] and 40 g/L sucrose, pH 5.7) to an OD600 of 0.5. The inoculum was delivered to the lamina tissue of *N. benthamiana* leaves by gentle pressure infiltration through the lower epidermis. To enhance transient expression of BiFC fusion proteins, the P19 viral suppressor of gene silencing was coexpressed [66]. YFP fluorescence was detected three days after infiltration. Tissue was mounted in low-melting-point agarose (0.4% in water) and viewed directly using an inverted Zeiss Observer Z1 spectral confocal laser microscope LSM 710 using a C-Apochromat ×63/1.20 W Corr objective (Carl Zeiss). Fluorescence was recorded after an excitation at 514 nm (Argon laser) and using a selective band of 514 to 568 nm.

### Complementation tests

A 5 kb *AtPSS1* genomic fragment containing 1.5 kb of promoter region and the complete *AtPSS1* gene was amplified using specific primers flanked by AttB1 and AttB2 sites (Table S2), cloned into Gateway vector pDONR207 using BP recombination (Invitrogen), and sequenced. Directed mutagenesis was performed using the Quickchange Site-Directed Mutagenesis Kit (Stratagene). The mutagenic primers used to generate the AtPSS1-R293H (Arg codon cgc→His codon cac) are shown in Table S2. A LR reaction between the resulting vectors and the pGWB1 destination binary vector was performed.

## Supporting Information

Figure S1 Pollen grain viability is affected in *Atpss1.* Alexander staining [68] of mature anthers. (A) wild type. All the pollen grains appear viable. (B) *Atpss1-1*. A significant proportion of the pollen grains are dead (∼30%). (C) Transformation of the *Atpss1-1* mutant with a 5 kb genomic region containing the *AtPSS1* gene restored pollen viability. Scale bar = 50 µm
(TIF)

Figure S2 Immunolocalization of ASY1 and ASY3 at leptotene in wild type and *Atpss1-1*. Cells were prepared according to Armstrong *et al.* [42]. Scale bar = 10 µm.
(TIF)

Figure S3 Female meiosis is affected in *Atpss1*. Ovules were prepared and stained with propidium iodide as decribed in Motamayor *et al.* [45]. (A) Wild-type ovule containing a meiocyte at metaphase I. Five bivalent are aligned on the metaphase plate. (B) An *Atpss1* ovule at the same stage. Two bivalents are aligned on the metaphase plate and six univalents are scattered in the meiocyte. b = bivalent, u = univalent. Scale bar = 10 µm.
(TIF)

Figure S4 Immunolocalization of HEI10 at diakinesis. Immunolocalization of HEI10 at diakinesis is shown (A) in wild type and (B) in *Atpss1-1*. (C, D) Scatter plot of HEI10 foci number per cell at diplotene and diakinesis. (E) Distribution of chromosomes according to their HEI10 foci number at diakinesis. Cells were prepared according to Chelysheva *et al.* [47]. Scale bar = 10 µm.
(TIF)

Figure S5 AtPSS1 and AtWIP1 interact in BiFC. *Nicotiana benthamiana* cells were infiltrated with different combinations of split YFP fusions with AtPSS1 and AtWIP1. (A) Co-expression of BiFC constructs YFP^N^-AtWIP1 and YFP^C^-AtPSS1 gave a clear cytoplasmic YFP fluorescence signal, revealing interaction between AtWIP1 and AtPSS1. (B, C) Negative controls correspond to co-expression of YFP^C^-AtPSS1 with the unrelated YFP^N^-GLOBOSA protein or YFP^N^-AtWIP1 with the unrelated YFP^C^-DEFICIENS protein. (D) Positive control corresponds to co-expression of YFP^N^-GLOBOSA with the YFP^C^-DEFICIENS protein. Scale bar = 50 µm.
(TIF)

Table S1 Tetrad analysis. (A) Tetrad raw data set. The FTL system relies on transgenic markers conferring cyan, yellow or red fluorescence of pollen grains within tetrads. Drawings above each column represent the different distribution possibilities of markers among the four chromatids and the corresponding distribution of colors in the tetrad, according to the nomenclature of Berchowitz and Copenhaver [46]. For each pair of intervals (e.g. I1b and I1c are two adjacent intervals on chromosome 1) and each genotype the observed number of each type of tetrad is given. (B) Interference analysis. Inter-interval interference was measured by comparing the genetic size of an interval (d, Perkins equation, cM) when a crossover occurs in an adjacent interval to the genetic size of the same interval when no crossover occurs in the adjacent interval. The ratio of these two distances, called the interference ratio (IR), gives a measurement of the strength of interference between two intervals [46] (e.g. IR^I2bI2a^ = (d(I2b) with CO in I2a)/(d(I2b) without CO in I2a)). The more this interference ratio is inferior to 1, the stronger interference is. Using the raw data from table S1A, calculations and statistical analyses have been performed according to Berchowitz and Copenhaver [46] and Stahl Lab Online tools (http://www.molbio.uoregon.edu/~fstahl/). For the three pairs of interval tested, genetic CO interference was detected in wild type (IR<1). In *Atpss1*, the IRs were not different from 1 and were statistically different from the wild-type IRs, showing that genetic CO interference is reduced or abolished in *Atpss1*.
(DOCX)

Table S2 PCR Primers used in this study.
(DOCX)
