# Supplementary figures and images for: The Kinesin AtPSS1 Promotes Synapsis and is Required for Proper Crossover Distribution in Meiosis
Source: PLoS Genet. 2014 Oct 16;10(10):e1004674. doi: 10.1371/journal.pgen.1004674 (PMC4199493; doi:10.1371/journal.pgen.1004674)

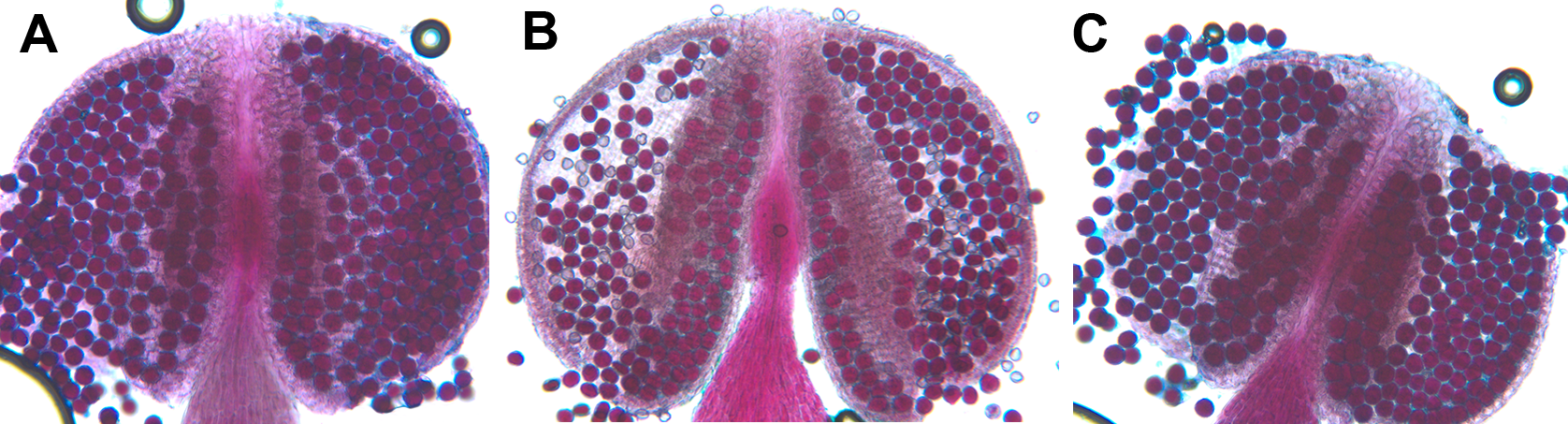

Supplement: Figure S1 — Pollen grain viability is affected in Atpss1. Alexander staining [68] of mature anthers. (A) wild type. All the pollen grains appear viable. (B) Atpss1-1. A significant proportion of the pollen grains are dead (∼30%). (C) Transformation of the Atpss1-1 mutant with a 5 kb genomic region containing the AtPSS1 gene restored pollen viability. Scale bar = 50 µm (TIF) [file pgen.1004674.s001.tif]

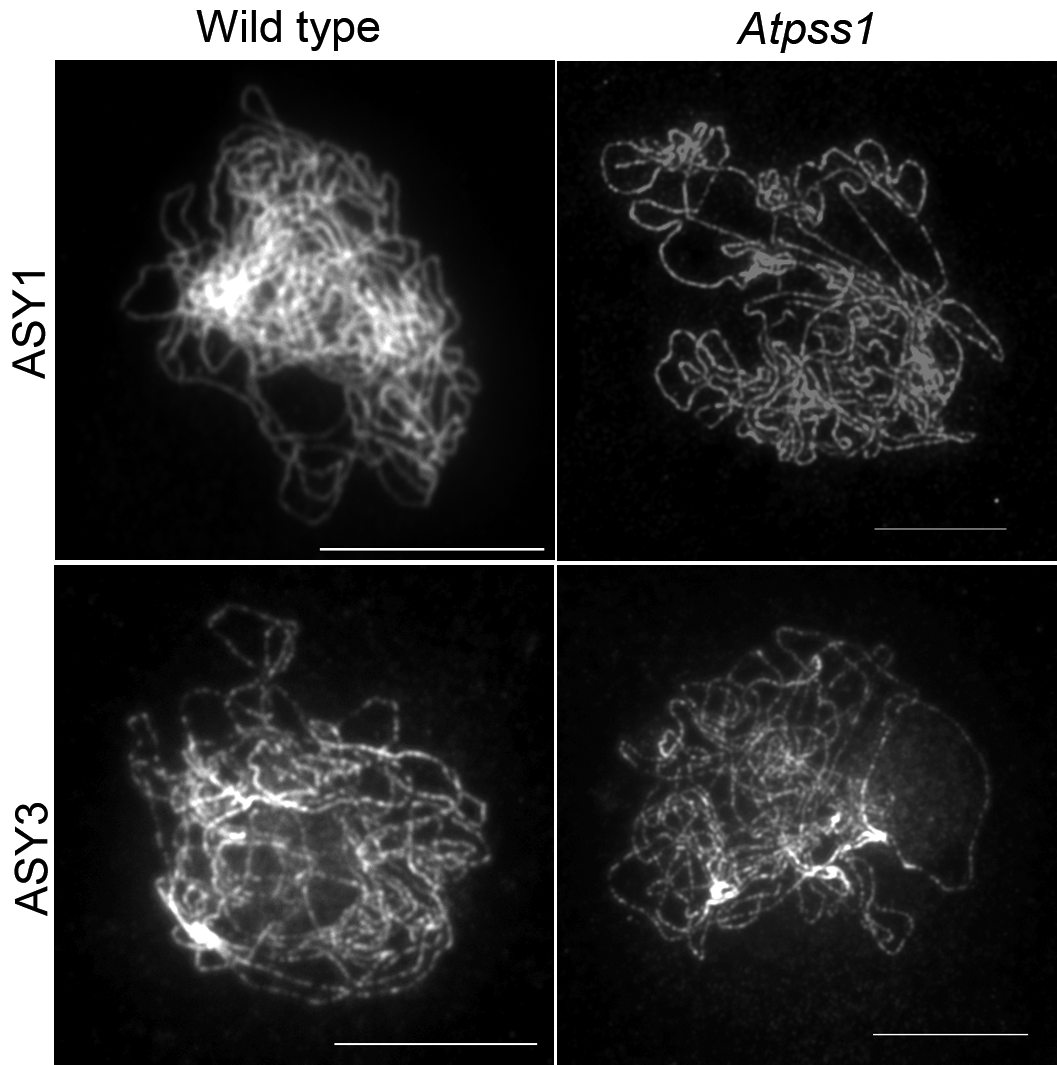

Supplement: Figure S2 — Immunolocalization of ASY1 and ASY3 at leptotene in wild type and Atpss1-1. Cells were prepared according to Armstrong et al. [42]. Scale bar = 10 µm. (TIF) [file pgen.1004674.s002.tif]

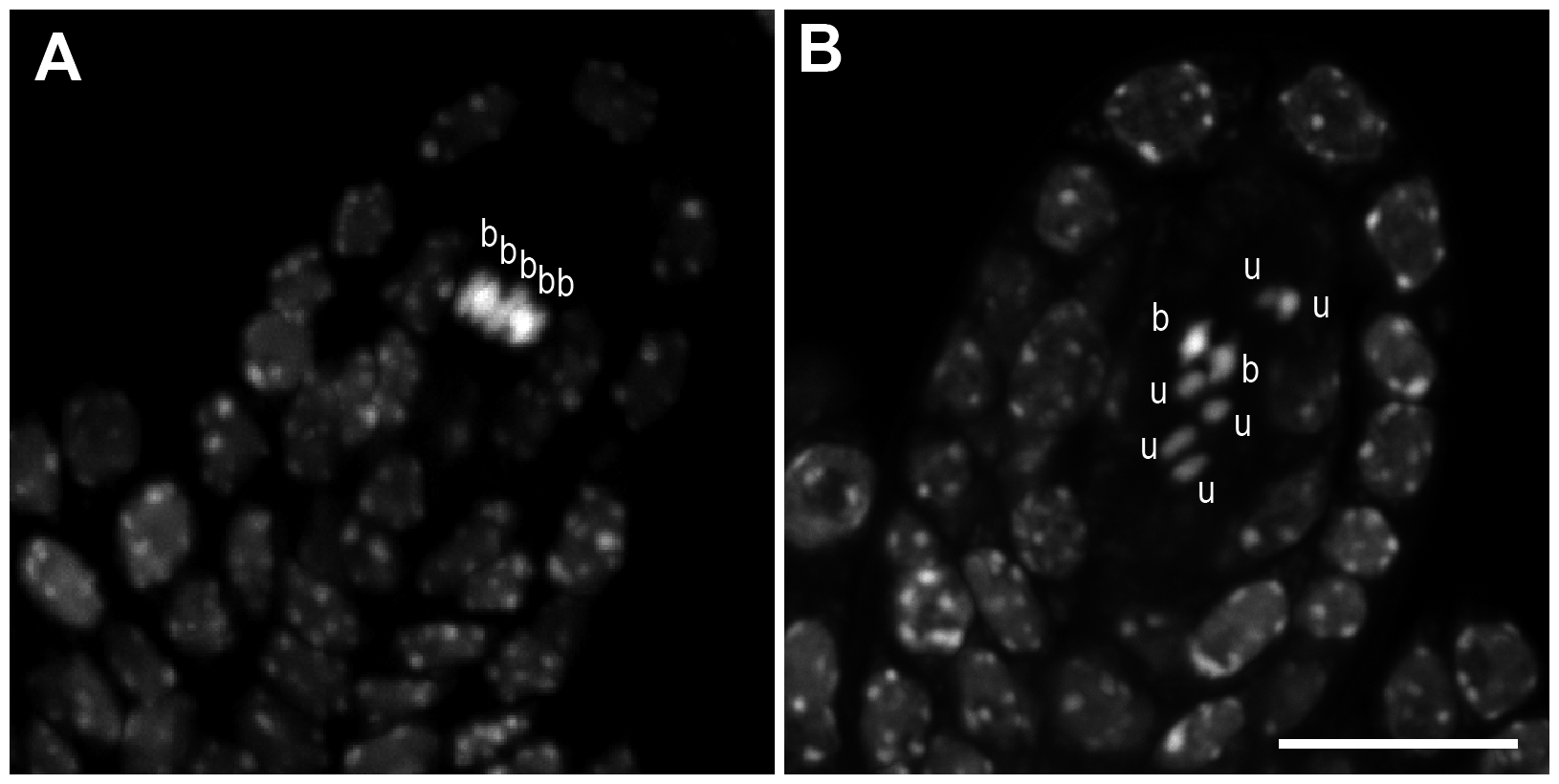

Supplement: Figure S3 — Female meiosis is affected in Atpss1. Ovules were prepared and stained with propidium iodide as decribed in Motamayor et al. [45]. (A) Wild-type ovule containing a meiocyte at metaphase I. Five bivalent are aligned on the metaphase plate. (B) An Atpss1 ovule at the same stage. Two bivalents are aligned on the metaphase plate and six univalents are scattered in the meiocyte. b = bivalent, u = univalent. Scale bar = 10 µm. (TIF) [file pgen.1004674.s003.tif]

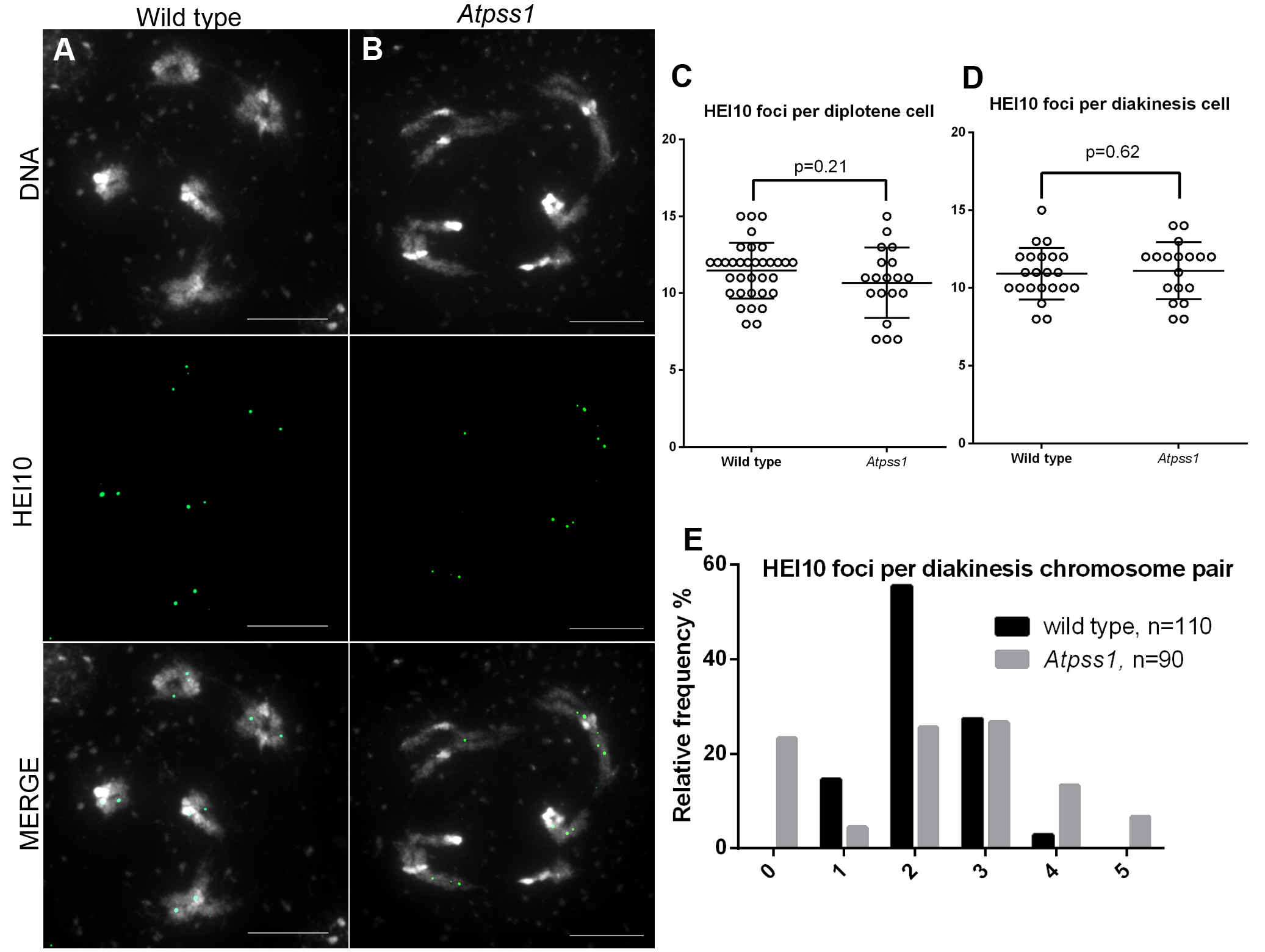

Supplement: Figure S4 — Immunolocalization of HEI10 at diakinesis. Immunolocalization of HEI10 at diakinesis is shown (A) in wild type and (B) in Atpss1-1. (C, D) Scatter plot of HEI10 foci number per cell at diplotene and diakinesis. (E) Distribution of chromosomes according to their HEI10 foci number at diakinesis. Cells were prepared according to Chelysheva et al. [47]. Scale bar = 10 µm. (TIF) [file pgen.1004674.s004.tif]

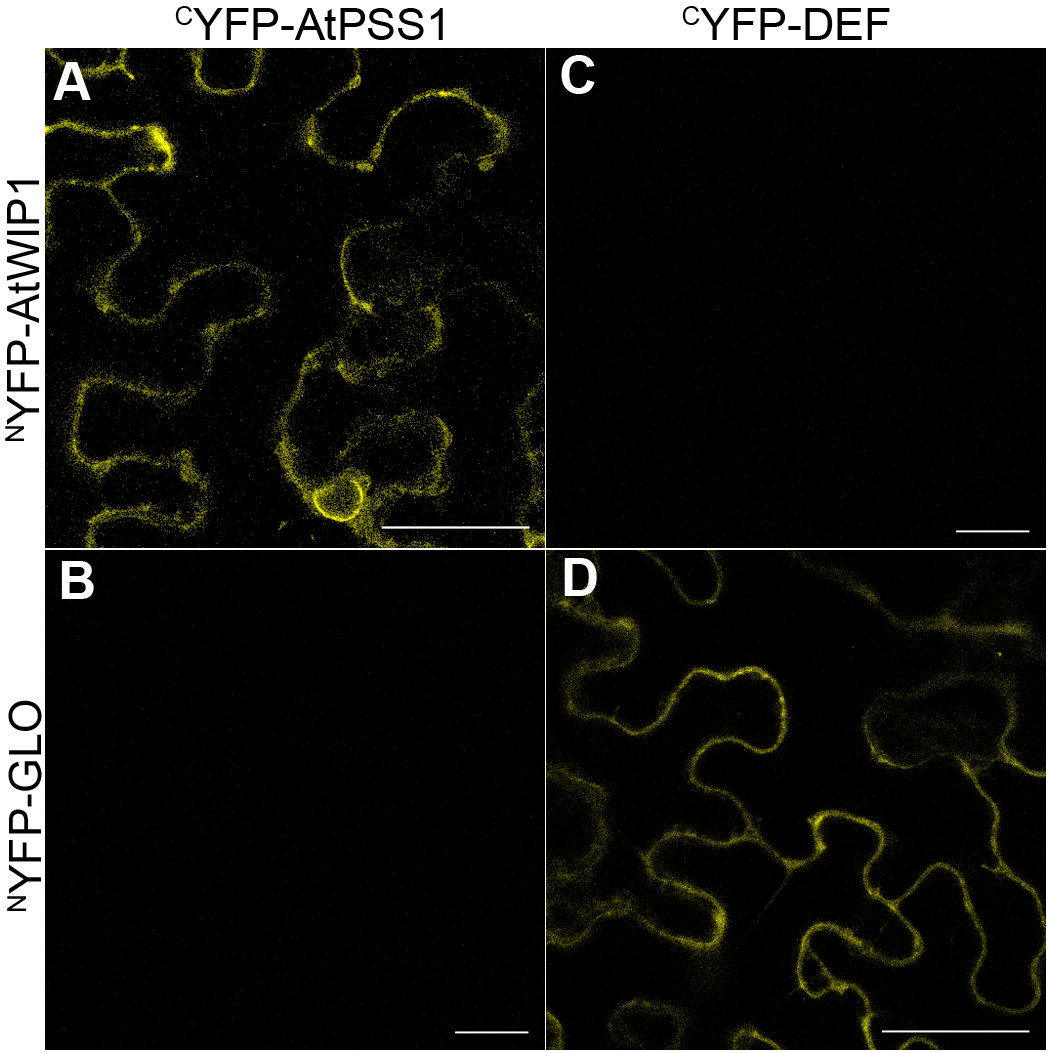

Supplement: Figure S5 — AtPSS1 and AtWIP1 interact in BiFC. Nicotiana benthamiana cells were infiltrated with different combinations of split YFP fusions with AtPSS1 and AtWIP1. (A) Co-expression of BiFC constructs YFPN-AtWIP1 and YFPC-AtPSS1 gave a clear cytoplasmic YFP fluorescence signal, revealing interaction between AtWIP1 and AtPSS1. (B, C) Negative controls correspond to co-expression of YFPC-AtPSS1 with the unrelated YFPN-GLOBOSA protein or YFPN-AtWIP1 with the unrelated YFPC-DEFICIENS protein. (D) Positive control corresponds to co-expression of YFPN-GLOBOSA with the YFPC-DEFICIENS protein. Scale bar = 50 µm. (TIF) [file pgen.1004674.s005.tif]
