# Supplementary material for: The Kinesin AtPSS1 Promotes Synapsis and is Required for Proper Crossover Distribution in Meiosis
Source: PLoS Genet. 2014 Oct 16;10(10):e1004674. doi: 10.1371/journal.pgen.1004674 (PMC4199493; doi:10.1371/journal.pgen.1004674)
Supplement: Table S1 — Tetrad analysis. (A) Tetrad raw data set. The FTL system relies on transgenic markers conferring cyan, yellow or red fluorescence of pollen grains within tetrads. Drawings above each column represent the different distribution possibilities of markers among the four chromatids and the corresponding distribution of colors in the tetrad, according to the nomenclature of Berchowitz and Copenhaver [46]. For each pair of intervals (e.g. I1b and I1c are two adjacent intervals on chromosome 1) and each genotype the observed number of each type of tetrad is given. (B) Interference analysis. Inter-interval interference was measured by comparing the genetic size of an interval (d, Perkins equation, cM) when a crossover occurs in an adjacent interval to the genetic size of the same interval when no crossover occurs in the adjacent interval. The ratio of these two distances, called the interference ratio (IR), gives a measurement of the strength of interference between two intervals [46] (e.g. IRI2bI2a = (d(I2b) with CO in I2a)/(d(I2b) without CO in I2a)). The more this interference ratio is inferior to 1, the stronger interference is. Using the raw data from table S1A, calculations and statistical analyses have been performed according to Berchowitz and Copenhaver [46] and Stahl Lab Online tools (http://www.molbio.uoregon.edu/~fstahl/). For the three pairs of interval tested, genetic CO interference was detected in wild type (IR<1). In Atpss1, the IRs were not different from 1 and were statistically different from the wild-type IRs, showing that genetic CO interference is reduced or abolished in Atpss1. (DOCX) [file pgen.1004674.s006.docx]

Table S1a. Recombination raw tetrad data set.

The FTL system relies on transgenic markers conferring cyan, yellow or red fluorescence of pollen grains within tetrads. Drawings above each column represent the different distribution possibilities of markers among the four chromatids and the corresponding distribution of colors in the tetrad, according to the nomenclature of Berchowitz and Copenhaver [1] *.* For each pair of intervals (*e.g.* I1bI1c are two adjacent intervals on chromosome 1) and each genotype the observed number of each type of tetrad is given.

­


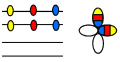

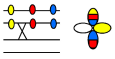

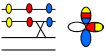

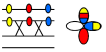

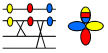

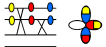

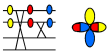

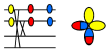

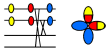

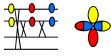

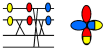

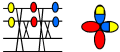


| **I1b I1c** | Total | a | b | c | d | e | F | g | h | i | j | k | l |
| --- | --- | --- | --- | --- | --- | --- | --- | --- | --- | --- | --- | --- | --- |
| wild type | 1527 | 800 | 185 | 493 | 5 | 12 | 13 | 9 | 0 | 9 | 0 | 1 | 0 |
| *Atpss1-1* | 1191 | 603 | 152 | 359 | 19 | 14 | 13 | 18 | 1 | 9 | 1 | 2 | 0 |
|  | 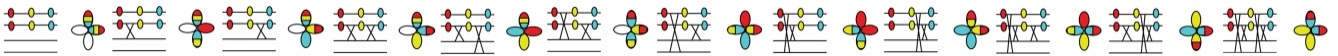 |  |  |  |  |  |  |  |  |  |  |  |  |
| **I2b I2a** | Total | a | b | c | d | e | F | g | h | i | j | K | l |
| wild type | 1778 | 1409 | 218 | 146 | 2 | 1 | 1 | 1 | 0 | 0 | 0 | 0 | 0 |
| *Atpss1-1* | 1230 | 915 | 216 | 80 | 3 | 7 | 3 | 2 | 4 | 0 | 0 | 0 | 0 |
|  | 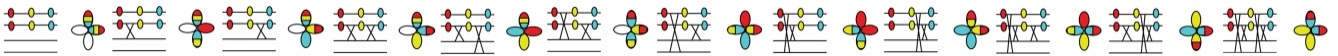 |  |  |  |  |  |  |  |  |  |  |  |  |
|  |  |  |  |  |  |  |  |  |  |  |  |  |  |
| **I5d I5c** | Total | a | b | c | d | e | F | g | h | i | j | k | l |
| wild type | 4030 | 3144 | 479 | 378 | 8 | 6 | 5 | 6 | 3 | 1 | 0 | 0 | 0 |
| *Atpss1-1* | 1680 | 1207 | 250 | 183 | 5 | 8 | 9 | 10 | 6 | 1 | 1 | 0 | 0 |

Table S1b. Interference analysis

Inter-interval interference was measured by comparing the genetic size of an interval (d, Perkins equation*,* cM) when a crossover occurs in an adjacent interval to the genetic size of the same interval when no crossover occurs in the adjacent interval. The ratio of these two distances, called the interference ratio (IR), gives a measurement of the strength of interference between two intervals [46] (e.g IR^I2bI2a^= (d(I2b) with CO in I2a)/ d(I2b) without CO in I2a)). The more this interference ratio is inferior to 1, the stronger interference is. Using the raw data from table S3, calculations and statistical analyses have been performed according to Berchowitz and Copenhaver [46] and Stahl Lab Online tools (http://www.molbio.uoregon.edu/~fstahl/).

For the three pairs of interval tested, genetic CO interference was detected in wild type (IR<1). In *Atpss1*, the IRs were not different from 1 and were statistically different from the wild type IRs, showing that genetic CO interference is reduced or abolished in *Atpss1*.

| **I2b I2a** | Wild type | *Atpss-1* |  | **I5d I5c** | Wild type | *Atpss1-1* |
| --- | --- | --- | --- | --- | --- | --- |
| d(I2b) without CO in I2a | 6.70 | 10.57 |  | d(I5d) without CO in I5c | 6.85 | 9.77 |
| d(I2b) with CO in I2a | 1.66 | 7.89 |  | d(I5d) with CO in I5c | 3.09 | 8.76 |
| Interference Ratio | 0.25 | 0.75 |  | Interference Ratio | 0.45 | 0.90 |
| p (IR=1) | <10^-5^ | 0.19 |  | p (IR=1) | <10^-5^ | 0.60 |
| p (IR=IR^wt^) | - | <10^-5^ |  | p (IR=IR^wt^) | - | 0.04 |
|  |  |  |  |  |  |  |
|  |  |  |  | **I1b I1c** | Wild type | *Atpss1-1* |
|  |  |  |  | d(I1b) without CO in I1c | 9.39 | 10.45 |
|  |  |  |  | d(I1b) with CO in I1c | 3.69 | 8.28 |
|  |  |  |  | Interference Ratio | 0.39 | 0.79 |
|  |  |  |  | p (IR=1) | <10^-5^ | 0.12 |
|  |  |  |  | p (IR=IR^wt^) | - | 0.004 |
