# Supplementary material for: The Kinesin AtPSS1 Promotes Synapsis and is Required for Proper Crossover Distribution in Meiosis
Source: PLoS Genet. 2014 Oct 16;10(10):e1004674. doi: 10.1371/journal.pgen.1004674 (PMC4199493; doi:10.1371/journal.pgen.1004674)
Supplement: Table S2 — PCR Primers used in this study. (DOCX) [file pgen.1004674.s007.docx]

|  |  |  | wild type alleles | | | mutant allele | | |
| --- | --- | --- | --- | --- | --- | --- | --- | --- |
| genotyping | gene | allele | Forward primer (Name / sequence) | Reverse primer (Name / sequence) | PCR conditions - amplicon size | Forward primer (Name / sequence) | Reverse primer (Name / sequence) | PCR conditions - amplicon size |
|  | Atpss1 | Atpps1-1 (WiscDsLox_343E05) | WDL343E05-LP / CTTCTTCAAGAACCCTACCTTCTG | WDL343E05-RP / CAGATTTTGATCTCAATTTGATCG | 60^1.5 -1200 | WDL343E05-RP / CAGATTTTGATCTCAATTTGATCG | P745 / AACGTCCGCAATGTGTTATTAAGTTGTC | 60^1 -600 |
|  |  | Atpps1-2 (SALK_120399) | SALK_120399-LP / AGGACTGTGATGAGCACAATAAAG | SALK_120399-RP / GCATGAGAGACCACAATAGAATTG | 57^1 - 1000 | SALK_120399-RP / GCATGAGAGACCACAATAGAATTG | Lbsalk2 / GCTTTCTTCCCTTCCTTTCTC | 57^1 - 750 |
|  |  | Atpps1-3 (SALK_024926) | SALK_024926-LP / TGGTATAAACGGAACAATCATCAC | SALK_024926-RP / CTGCTGGACATATTCATTTCTGTC | 57^1 - 1000 | SALK_024926-RP / CTGCTGGACATATTCATTTCTGTC | Lbsalk2 / GCTTTCTTCCCTTCCTTTCTC | 57^1 - 750 |
|  | zip4 | zip4-2 (SALK_068052) | EJD21-P5 / GACTGCTGGAGCAGAAACT | EJD21-P11 / CCATCTGAGAGCGAAGAAGAC | 60^1 - 1000 | EJD21-PWI / ATCTTGAACTGCAAGAATCA | Lbsalk2 / GCTTTCTTCCCTTCCTTTCTC | 60^1.5 - 1500 |
|  | msh5 | msh5-2 (SALK_026553) | N526553 U / ACATGGCTTGTATTCAGCAC | N526553 L / CAATCAGTGTAAAACAGCAGG | 60^1 - 900 | N526553 L / CAATCAGTGTAAAACAGCAGG | Lbsalk2 / GCTTTCTTCCCTTCCTTTCTC | 60^1 -750 |
|  | mus81 | mus81-2 (SALK_107515) | N607515U / CATGCTGACAGTTGAAGGTC | N607515L / CCTCAAACGTTTCTCCAAAT | 60^1 - 1000 | N607515L / CCTCAAACGTTTCTCCAAAT | Lbsalk2 / GCTTTCTTCCCTTCCTTTCTC | 60^1 -750 |
|  | fancm | fancm-1 | At1g35530dCAPSF1 / ACAATATATGTTTCGTGCAGGTAAGACATTGGAAG | At1g35530dCAPSR1 / CACCAATAGATGTTGCGACAAT | 57^0.5 - 220 | Digestion by MboII / | / |  |
|  | sun1 | SAIL_84_G10 | SUN1-G10-F / GGGGTTATTTCAATGACAATAACCGAG | SUN1-G10-R / GATGCGTTTTAAAGATTAACAGTATAAATTGG | 57^1 - 900 | SUN1-G10-R / GATGCGTTTTAAAGATTAACAGTATAAATTGG | LB1 / GCCTTTTCAGAAATGGATAAATAGCCTTGCTTCC | 57^1 - 600 |
|  | sun2 | FLAG_026E12 | SUN2-FLAG-F1 / GCTGTGACAATATGCATTGAGGAGG | SUN2-FLAG-R1 / GACTGAGTCTAGTTCACGGCC | 57^1 - 800 | SUN2-FLAG-F1 / GCTGTGACAATATGCATTGAGGAGG | FLAG-LB4 / GCCAGGTGCCCACGGATAGT | 57^1 - 600 |
|  |  |  |  |  |  |  |  |  |
| cloning / mutagenesis | gene | primer F | sequence | | primer R | sequence | | |
|  | Atpss1 (cds) | PSS1-cds-GW-U | GGGGACAAGTTTGTACAAAAAAGCAGGCTTGATGTCTAACGTAACCGTCTGTGCGCGATT | | PSS1-cds-GW-L-stop | GGGGACCACTTTGTACAAGAAAGCTGGGTATTAGGACGTAAAGAACGATGCATACCA | | |
|  |  |  |  |  | PSS1-cds-GW-L-nostop | GGGGACCACTTTGTACAAGAAAGCTGGGTAGGACGTAAAGAACGATGCATACCAAGAAC | | |
|  | Atpss1 (genomic) | PSS1-GW-U | GGGGACAAGTTTGTACAAAAAAGCAGGCTTGTGGATACATATTACCTCGA | | PSS1-GW-L | GGGGACCACTTTGTACAAGAAAGCTGGGTATGTTGGAGCTCTCTTTCTTT | | |
|  | Atpss1-R293H (genomic) | PSS1-R293H-U | CCATATCGTGACTCCAAGCTTACTCACATCTTACAGGATGCCCTGGTTAGT | | PSS1-R293H-L | ACTAACCAGGGCATCCTGTAAGATGTGAGTAAGCTTGGAGTCACGATATGG | | |
|  | Sun1 | SUN-GW-U | GGGGACAAGTTTGTACAAAAAAGCAGGCTTGATGTCGGCGTCAACGGTGTCAATC | | SUN1-cds-GW-L-stop | GGGGACCACTTTGTACAAGAAAGCTGGGTATTATTCACTTTCAGGTGAAGAGTCC | | |
|  |  |  |  |  | SUN1-cds-GW-L-nostop | GGGGACCACTTTGTACAAGAAAGCTGGGTATCAGGACTCTTCACCTGAAAGTGAA | | |
|  | Sun2 | SUN-GW-U | GGGGACAAGTTTGTACAAAAAAGCAGGCTTGATGTCGGCGTCAACGGTGTCAATC | | SUN2-cds-GW-L-stop | GGGGACCACTTTGTACAAGAAAGCTGGGTATCAAGCATGAGCAACAGAGACTGAG | | |
|  |  |  |  |  | SUN2-cds-GW-L-nostop | GGGGACCACTTTGTACAAGAAAGCTGGGTAAGCATGAGCAACAGAGACTGAGTCT | | |
|  | wip1 | WIP1-GW-U | GGGGACAAGTTTGTACAAAAAAGCAGGCTTGATGGATTTGGAGAGTGAAAGCTCTGCAC | | WIP1-cds-GW-L-stop | GGGGACCACTTTGTACAAGAAAGCTGGGTATCATGTGGGTACAACAGTATCTGGCTCC | | |
|  |  |  |  |  | WIP1-cds-GW-L-nostop | GGGGACCACTTTGTACAAGAAAGCTGGGTATGTGGGTACAACAGTATCTGGCTCCGGC | | |
|  | wip2 | WIP2-GW-U | GGGGACAAGTTTGTACAAAAAAGCAGGCTTGATGGATTTGGAGAGTGAAAGCTCTGTTC | | WIP2-cds-GW-L-stop | GGGGACCACTTTGTACAAGAAAGCTGGGTATCATGTGGGAACAGCAATCTCCGGTTCT | | |
|  |  |  |  |  | WIP2-cds-GW-L-nostop | GGGGACCACTTTGTACAAGAAAGCTGGGTATGTGGGAACAGCAATCTCCGGTTCTGGT | | |
|  | wip3 | WIP3-GW-U | GGGGACAAGTTTGTACAAAAAAGCAGGCTTGATGAATGAGTCAGTCCCTGATTCTGTGG | | WIP3-cds-GW-L-stop | GGGGACCACTTTGTACAAGAAAGCTGGGTATCAAGTTGGTATTACTAATCGTGAAGCA | | |
|  |  |  |  |  | WIP3-cds-GW-L-nostop | GGGGACCACTTTGTACAAGAAAGCTGGGTAAGTTGGTATTACTAATCGTGAAGCAGGA | | |
